# An Evaluation of Whether Routine QTc Interval Screening Is Necessary Prior to Starting ADHD Medications: Experience from a Large Retrospective Study

**DOI:** 10.3390/pediatric16040098

**Published:** 2024-12-11

**Authors:** Hamza A. Alsayouf, Lima M. Dyab, Redab Al-Ghawanmeh, Luay S. Alhawawsha, Osama Alsarhan, Hadeel Al-Smadi, Ghaith M. Al-Taani, Azhar Daoud, Haitham E. Elsadek, Wael H. Khreisat

**Affiliations:** 1 Dr Hamza Alsayouf Clinic, Amman 11181, Jordan; dr.osama91alsarhan@gmail.com (O.A.); dr_hytham3000@yahoo.com (H.E.E.); 2 Arab Medical Center, Amman 11181, Jordan; limadyab@gmail.com; 3 Department of Pediatrics, Faculty of Medicine, The Hashemite University, Zarqa 13133, Jordan; redab@hu.edu.jo; 4 Royal Medical Services, Amman 11855, Jordan; loai_09@yahoo.com; 5 Faculty of Medicine, Yarmouk University, Irbid 21163, Jordan; hadeelsmadi565@gmail.com; 6 Department of Clinical Pharmacy and Pharmacy Practice, Faculty of Pharmacy, Yarmouk University, Irbid 21163, Jordan; g.altaani@yu.edu.jo; 7 The Specialty Hospital, Amman 11942, Jordan; azhardaoud@gmail.com; 8 Department of Pediatric Neurology, Queen Rania Children’s Hospital, Royal Medical Services, Amman 11118, Jordan; wael_khreisat@yahoo.com

**Keywords:** aripiprazole, attention-deficit/hyperactivity disorder, atypical antipsychotics, electrocardiograms, risperidone, QTc interval

## Abstract

Background/Objectives: Routine screening electrocardiograms (ECGs) prior to starting medications for attention-deficit/hyperactivity disorder (ADHD) remain controversial. This real-world study assessed corrected QT (QTc) interval data from pediatric patients who had a baseline ECG performed prior to initiating treatment with ADHD medications and ≥6 months of clinical follow-up. Methods: A retrospective chart review of children aged 2–18 years diagnosed with ADHD with/without autism spectrum disorder (ASD) at child neurology clinics in Jordan (June 2019 and June 2021) was performed, and children were prescribed with ADHD medications to manage symptoms. Patients had ≥6 months of follow-up and no known cardiac disease/family history. A baseline ECG and regular clinical exams were performed for each child. Results: Of 458 patients with baseline ECGs, 362 met the study inclusion criteria. Overall, 286 (79.0%) patients were diagnosed with ASD/comorbid ADHD and 76 (21.0%) with ADHD alone; 61 (16.9%) were prescribed atomoxetine, 38 (10.5%) methylphenidate, 134 (37.0%) risperidone, and 129 (35.6%) aripiprazole. The patients’ mean ± SD age was 6.4 ± 3.5 years, and most were male (n = 268, 74.0%). The mean baseline QTc interval was 400 ± 22 ms (median, 400 ms); one patient had a QTc interval >460 ms and was excluded from initiating treatment with any ADHD medications. During the ≥6-month follow-up, none of the patients had any signs or symptoms of adverse cardiac effects. Conclusions: Routine screening ECGs prior to treatment with ADHD medications may not be necessary in healthy children with no family history of cardiac disease. However, further studies are needed to evaluate the long-term effects of ADHD medications in low-risk pediatric patients.

## 1. Introduction

Attention-deficit/hyperactivity disorder (ADHD) is a common neurodevelopmental disorder that often manifests in childhood and can persist into adulthood [1]. Children with ADHD may struggle with attention and impulsivity, and they typically exhibit high levels of activity. This disorder presents challenges in various aspects of daily life, affecting individuals’ functioning in a variety of settings [1].

There is ongoing debate regarding the over-prescription of ADHD medications, as well as concerns about their potential for abuse and long-term adverse effects, particularly among children [2,3]. Despite this controversy, medications remain a widely used treatment option for managing ADHD symptoms, including in patients with comorbid autism spectrum disorder (ASD) [2]. Regulatory authorities and clinical guidelines on the management of ADHD have raised concerns about the safety of the medications used and the need for thorough pre-treatment assessments [4,5,6]. With regard to cardiovascular safety, the consensus to date is that while severe cardiovascular incidents related to ADHD medications are rare, healthcare providers must monitor patients for signs of cardiac effects, and collaboration among healthcare professionals, patients, and families is crucial for safe ADHD management [7,8].

The routine use of screening electrocardiograms (ECGs) prior to starting medications for ADHD remains controversial [9]. A 2008 statement by the American Heart Association recommended routine screening with ECGs to test for cardiac conditions in children with ADHD prior to being treated with ADHD medications [6]. In contrast, the American Academy of Child and Adolescent Psychiatry and the American Academy of Pediatrics concluded that there was a lack of evidence supporting routine ECGs [5], and the 2009 joint position statement by the Canadian Paediatric Society, Canadian Cardiovascular Society, and Canadian Academy of Child and Adolescent Psychiatry similarly did not support routine ECGs prior to initiating ADHD medications [10]. Instead, these bodies recommended that clinicians with ADHD expertise prescribe medications at their own discretion to patients with heart disease, while acknowledging the importance of monitoring by a cardiologist.

Although collaboration between ADHD experts and cardiologists represents the ideal scenario, in many regions, this is only available to physicians working in tertiary centers. Furthermore, most primary care physicians working in rural areas and developing countries have very limited access to well-trained pediatric cardiologists [10].

In this retrospective study, we aimed to assess whether routine screening ECGs provided a necessary benefit in a cohort of children prior to initiating treatment with ADHD medications. These medications included the stimulant methylphenidate, the norepinephrine reuptake inhibitor atomoxetine, and the atypical antipsychotics, aripiprazole and risperidone. The findings of this study suggest that routine screening ECGs may not be necessary prior to starting treatment with ADHD medications in healthy children with no family history of cardiac disease.

## 2. Materials and Methods

We conducted a retrospective chart review of all children who were diagnosed with ADHD with/without comorbid ASD at the child neurology clinics of the authors in Jordan between June 2019 and June 2021, and the children were prescribed treatment with aripiprazole, atomoxetine, methylphenidate, or risperidone to manage their ADHD symptoms. ADHD and ASD diagnoses were determined by a child neurologist and psychologist on the basis of a structured interview using DSM-5 criteria [11].

The inclusion criteria for this study were as follows: (1) diagnosis of ADHD (with or without comorbid ASD), (2) age of 2 to 18 years, (3) not known to have any cardiac disease, (4) no significant family history of sudden death or cardiac disease early in life, (5) compliance with follow-up for at least 6 months, (6) not taking any other ADHD medications or medications that could affect the corrected QT (QTc) interval at baseline or during treatment at our clinic, and (7) the patient’s family consented to the treatment plan.

Verbal assent and written consent were obtained from patients and their families before initiating treatment. This study was approved by the independent Pearl Institutional Review Board (IRB) as an exemption (IRB approval number: 20-KNRC-102) and followed the ethical standards of the 2000 revision of the 1975 Declaration of Helsinki.

For each child attending our institution, we reviewed data regarding their age, sex, and current and previous medications. Any significant personal medical history was recorded, including any cardiac signs and symptoms such as palpitations, dyspnea, fatigue, chest pain, or syncope. In addition, a family history was taken with specific questions regarding cases of sudden death, cardiovascular symptoms, Wolff–Parkinson–White syndrome, hypertrophic cardiomyopathy, and long QT syndrome.

A baseline ECG was performed for each patient, with a particular focus on the QTc interval. The QTc interval was calculated manually by a pediatric cardiologist using the Bazett formula [12]. A clinical exam was performed at the first visit and each of the follow-up visits, during which the patient’s heart rate (HR), respiratory rate (RR), blood pressure (BP), weight, and height were measured.

## 3. Results

**Figure 1:**
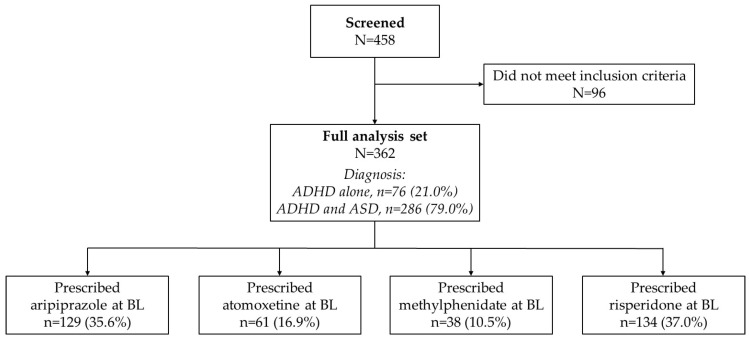
Patient disposition. ADHD, attention-deficit/hyperactivity disorder; ASD, autism spectrum disorder; BL, baseline.

A total of 458 patients had screening ECGs performed at baseline at the authors’ clinics during the study period. After excluding those who did not meet the inclusion criteria, 362 patients remained. Of these, 286 (79.0%) patients met the criteria for ASD with comorbid ADHD and 76 (21.0%) met the diagnostic criteria for ADHD alone; 61 (16.9%) patients had been prescribed with atomoxetine, 38 (10.5%) patients with methylphenidate, 134 (37.0%) patients with risperidone, and 129 (35.6%) with aripiprazole (Figure 1).

The majority of patients were male (n = 268, 74.0%), and their ages ranged from 2 to 18 years with a mean ± standard deviation (SD) of 6.4 ± 3.5 years (median, 6 years); Table 1. Of the 362 patients, 31 were aged ≥ 12 years, indicating late diagnosis and treatment.

The baseline QTc interval in these patients ranged from 341 ms to a maximum value of 465 ms; only one patient had a QTc interval above 460 ms, and we excluded this patient from starting treatment with any ADHD medications. The mean ± SD QTc interval was 400 ± 22 ms (median, 400 ms).

During their ≥6-month follow-up, none of the patients had any signs or symptoms of drug-related cardiac side effects such as tachycardia, chest pain, palpitations, dyspnea, abnormal breathing patterns, or fatigue, and all patients were able to continue with treatment.

## 4. Discussion

This retrospective cohort study evaluated the QTc interval in 362 patients aged 2–18 years with ASD and/or ADHD who were otherwise healthy with no family history of cardiac disease. For each of these patients, an ECG was performed prior to starting treatment with ADHD medications. The medications prescribed for ADHD treatment included the stimulant methylphenidate, the norepinephrine reuptake inhibitor atomoxetine, and the atypical antipsychotics, aripiprazole and risperidone. In addition to an ECG at baseline, patients were assessed at baseline and at subsequent follow-up visits for any signs or symptoms of cardiac effects. These assessments aimed to provide a comprehensive understanding of the potential effects of ADHD medications on cardiac parameters, thereby informing clinical decision-making and patient management.

Our ≥6-month clinical follow-up of these patients did not indicate any negative cardiac effects of these medications in this cohort. Baseline ECG screening helped to exclude one patient who had a QTc interval greater than 460 ms, for whom we considered it a risk to initiate treatment. In children 1–15 years of age, a QTc of between 440 and 460 ms is considered the upper limit of a normal QTc interval, whereas a QTc of >460 ms is considered prolonged [13].

The main reason to measure the QTc interval at screening is to exclude patients with long QT syndrome, as these patients are considered high risk for cardiac adverse events during treatment with ADHD medications [14]. The QT interval signifies the duration from the initiation of ventricular depolarization to the completion of repolarization; it aligns with the timeframe for mechanical systole, and it changes in response to fluctuations in the HR [15]. However, in long QT syndrome, cardiac adaptation to HR changes is disturbed, potentially leading to arrhythmias or even sudden death.

Stimulant medications may result in a slight elevation in a patient’s HR and BP on average [9,16,17]. However, it is important to note that in a subset of children and adolescents (approximately 5–15%), stimulants may lead to more pronounced elevations in HR and BP [18]. Several studies have aimed to assess the frequency of cardiovascular incidents in children and young adults using stimulant medications for ADHD. For example, in a study of 55,383 patients with ADHD aged 3 to 20 years, the rates of cardiac events requiring hospitalization were small and similar to national background rates, although stimulants were associated with an increase in cardiac emergency department visits [19]. In contrast, in a population-based cohort study involving over 1.2 million children aged 3 to 18 years, no increase was observed in the risk of severe cardiovascular incidents during treatment with stimulants [20]; comparable findings were obtained from a large analysis of claims data from patients with ADHD or ASD aged 3 to 18 years in the USA [16]. Furthermore, a meta-analysis of ADHD medication trials (including studies of stimulants) in children found that while 2% of children discontinued treatment due to cardiovascular effects, no serious cardiovascular events were reported [17].

Notably, the history of stimulants in ADHD treatment has been complex and controversial due to changes in amphetamine salts [21] and the inclusion of black-box warnings by regulatory agencies such as the USA’s Food and Drug Administration (FDA) [5,10]. Clinicians are advised to closely monitor vital signs at each visit in patients undergoing stimulant treatment; in addition, prior to commencing treatment with stimulant medications, it is crucial to gather the patient’s medical history, focusing on any specific cardiac symptoms they may have experienced [5,7,10]. Furthermore, a family history needs to be obtained of sudden death, cardiovascular symptoms, Wolff–Parkinson–White syndrome, hypertrophic cardiomyopathy, and long QT syndrome. If any of these risk factors are identified, clinicians should conduct a further evaluation to assess and manage potential safety concerns associated with the use of stimulant medications, preferably at a tertiary center.

Non-stimulant ADHD medications such as atomoxetine can be used for those patients who cannot tolerate stimulant therapy or otherwise require an alternative to stimulant medications [22]. However, atomoxetine works by boosting norepinephrine levels, thereby potentially raising HR and BP [16,17,22]. With atomoxetine, significant improvements in ADHD symptoms can be observed within weeks; patients may experience enhanced attention, focus, and impulse control with this therapy [23,24], although monitoring for side effects and adjusting dosage as necessary is critical to optimize treatment outcomes [22]. The FDA has warned of a higher suicide risk in children on atomoxetine vs. placebo [25]. However, no evidence of an increased risk of suicide-related events has been observed in large, real-world studies of atomoxetine in children [26,27]; instead, it has been suggested that ADHD medications such as atomoxetine and methylphenidate may have a protective effect against such events [26,28]. It is important to note that other factors such as depression and ADHD itself may also contribute to suicide risk in children [29].

In contrast to first-generation antipsychotics, second-generation antipsychotics such as risperidone and aripiprazole do not appear to be associated with serious or clinically relevant cardiac effects. In a study of 105 adult patients by Dodd et al., no link was found between the prolongation of QTc interval dispersion and the use of medications such as atypical antipsychotics [30]. In an open-label prospective study in children aged 4 to 15 years, aripiprazole and risperidone treatment for various psychiatric conditions was not associated with clinically relevant changes in the QT interval [31]. Similarly, a long-term retrospective study in 101 children treated with antipsychotics (mostly risperidone and aripiprazole) reported seven children with changes in their QTc interval but all were asymptomatic with a QTc of <500 ms [32]. In a retrospective study of 804 adult psychiatric inpatients, no association was found between drug serum concentrations within the therapeutic range and prolongation of the QTc interval for aripiprazole or risperidone after correction for known genetic factors and the number of potential QT-prolonging drugs administered [33].

The 2009 recommendations from the American Heart Association [6] sparked significant debate [5,10,34,35]. Members of the American Academy of Pediatrics subsequently expressed concerns that the potential harm of recommending routine ECGs for healthy children starting stimulant medication for ADHD may outweigh the benefits [36]. They also questioned the accuracy of ECGs as a general screening tool, particularly highlighting the risk of a high number of false positive results. Furthermore, since Bazett first proposed a QTc correction formula in 1920 (QTc = QT/√RR) [12], many variations of this formula have become available; all these formulae have limitations and may not accurately reflect QT interval changes in all individuals. Finally, it is important to consider the cost-effectiveness and practicality of implementing routine baseline ECG screening for all patients with ADHD [6], especially in rural areas where this may not be available and given that ECGs can be challenging to perform in children with comorbid ASD (some patients may even require sedation) [37,38]. Indeed, the stress and potential psychological impact on young patients undergoing ECG screening should be carefully considered. However, it is clear that for children with ADHD and comorbid heart disease, close monitoring and communication between healthcare providers are essential to ensure patient safety [7,8,10].

## 5. Conclusions

The findings of this retrospective cohort study suggest that routine screening ECGs may not be necessary prior to starting treatment with ADHD medications in healthy children with no family history of cardiac disease. However, further research and long-term prospective studies are needed to fully understand the potential impact of ADHD medications on heart health in low-risk pediatric patients.

## Figures and Tables

**Table 1:** Baseline demographics and characteristics.

Baseline Demographic and Disease Characteristics	Full Analysis Set (N = 362)
Sex, n (%)	
Male	268 (74.0)
Female	94 (26.0)
Age, years	
Mean ± SD	6.4 ± 3.5
Median	6.0
QTc interval, ms	
Mean ± SD	400 ± 22
Median	400
Min, max	341, 465

Min, minimum; max, maximum; SD, standard deviation; QTc, corrected QT interval.

## Data Availability

The data presented in this study are available upon reasonable request from the corresponding author due to privacy and ethical restrictions.
